# Using Technology to Identify Children With Autism Through Motor Abnormalities

**DOI:** 10.3389/fpsyg.2021.635696

**Published:** 2021-05-25

**Authors:** Roberta Simeoli, Nicola Milano, Angelo Rega, Davide Marocco

**Affiliations:** ^1^Department of Humanistic Studies, University of Naples Federico II, Naples, Italy; ^2^Institute of Cognitive Sciences and Technologies, National Research Council (CNR), Rome, Italy; ^3^Neapolisanit S.R.L. Rehabilitation Center, Ottaviano, Italy

**Keywords:** autism spectrum disorder, sensory-motor impairment, motion analysis, machine learning, classification, assessment technologies

## Abstract

Autism is a neurodevelopmental disorder typically assessed and diagnosed through observational analysis of behavior. Assessment exclusively based on behavioral observation sessions requires a lot of time for the diagnosis. In recent years, there is a growing need to make assessment processes more motivating and capable to provide objective measures of the disorder. New evidence showed that motor abnormalities may underpin the disorder and provide a computational marker to enhance assessment and diagnostic processes. Thus, a measure of motor patterns could provide a means to assess young children with autism and a new starting point for rehabilitation treatments. In this study, we propose to use a software tool that through a smart tablet device and touch screen sensor technologies could be able to capture detailed information about children’s motor patterns. We compared movement trajectories of autistic children and typically developing children, with the aim to identify autism motor signatures analyzing their coordinates of movements. We used a smart tablet device to record coordinates of dragging movements carried out by 60 children (30 autistic children and 30 typically developing children) during a cognitive task. Machine learning analysis of children’s motor patterns identified autism with 93% accuracy, demonstrating that autism can be computationally identified. The analysis of the features that most affect the prediction reveals and describes the differences between the groups, confirming that motor abnormalities are a core feature of autism.

## Introduction

Autism spectrum disorder (ASD) is a neurodevelopmental disorder notoriously characterized by communication impairment, a lack of social interaction, and the presence of restricted, repetitive, and stereotyped behaviors. Clinically, autism includes a very variable repertoire of symptoms and manifestations. The different target behaviors occur for each child with different degrees of severity. The etiology of the disorder is still unknown, and it can involve both genetic and environmental factors. Because of these variabilities, many specialists are assuming that autism can be classified into different types, each of which may have different etiology and response to treatment. Recent studies have defined ASD as the most frequently observed neurodevelopmental disorder with an incidence estimate of 60–70/10,000 (Fombonne, 2009). The scientific community is currently moving in the direction of deepening the etiopathogenesis of the disorder and increasingly refine the techniques of diagnosis and assessment (Bertoglio and Hendren, 2009; Fombonne, 2009; Lenoir et al., 2009). Assuming that the disorder could be influenced by both genetic and environmental factors (Hallmayer et al., 2011; Sandin et al., 2014), intervention on this second aspect becomes a core topic for clinicians. An effective environmental intervention is possible through early and targeted therapeutic treatments. For these reasons, an early diagnosis becomes a fundamental step to set up a more effective therapeutic intervention (Howlin et al., 2009; Bradshaw et al., 2015). The diagnosis of autism is recommended from 3 years of age by the Roehr (2013), and this is due to the most widely used diagnostic tools and the only ones to be validated that are based on observational analysis of the specific behaviors considered the core symptoms of the disorder according to the Diagnostic and Statistical Manual of Mental Disorders (5th ed.). These symptoms mainly concern the communication area, social interaction, and the presence of ritualistic, repetitive, and stereotyped behaviors. Heretofore, the diagnostic tools relied on the interpretative skills of clinicians during the administration of paper and pencil tests and the support of parents and caregivers who provided salient information through structured interviews. All these methods take a long time without being able to deliver an objective and shareable result. One of the main gaps in the diagnosis of autism is related to the lack of a quantitative evaluation of the disorder; in fact, although the variability within the disorder is known, there is still no valid method to recognize and categorize these differences. These limits increase the time to diagnosis due to the uncertainty in the clinical diagnostic fit. In recent years, the literature on ASD has been focusing on identifying the links between the core symptoms at a high level and the corresponding impairment at a lower level. In the perspective of embodied cognition, these behavioral anomalies or, more generally, these high-level cognitive dysfunctions cease to be considered the central focus of the syndrome and begin to be analyzed as mere manifestations of underlying physiological dysfunctions and neural abnormalities. It would mean that ASD individuals cope with dysfunctions present at much lower levels, not only at the level of the central nervous system but also at the one of the peripheral nervous system and autonomic nervous system (Torres et al., 2013).

Kanner (1943) was the first to identify the disruption of normal movement patterns as a cardinal feature of ASD. Leary and Hill (1996) were among the first to identify a link between motor disorder and autistic symptoms, focusing on the effects of motor abnormalities on language, emotional expressions, and social interaction. This new point of view was followed by consideration of an “enactive mind” approach (Klin et al., 2003), according to which “… social cognitive processes emerge only from recurrent sensorimotor patterns that allow action to be perceptually guided.”

In 2014, Friston recognized the presence of anomalies in the predictive coding systems associated with ASD. This anomaly originates from perceptual systems and from an impairment of the integration of sensory information that would lead to maladaptive motor acts. In this scenario, it is difficult to identify and topographically define the resulting motor anomaly. In the last years, several studies aimed to identify the specific characteristics of motor abnormalities in autism.

Frequently, ASD is associated with greater clumsiness, motor coordination abnormalities, postural instability, and abnormalities in the kinematics of purposeful movements, such as grasping, reaching, or writing (Bauman, 1992; Ghaziuddin et al., 1994; Molloy et al., 2003; Dowd et al., 2012; Sacrey et al., 2014; Stoit et al., 2013; Kushki et al., 2011). Many studies identify movement abnormalities during prospective, goal-directed motor control (Trevarthen and Delafield-Butt, 2013) and an ineffective prospective organization during a series or chains of movements (Fabbri-Destro et al., 2009).

An interesting interpretative proposal was given by Sinha et al. (2014), and the authors focused on the predictive abilities of individuals with autism, to explain their abnormal behaviors, “…if our predictive abilities were somehow to be compromised, then even mundane occurrences in the environment might appear magical… A magical world suggests lack of control and impairs one’s ability to take preparatory actions. It can result in outcomes such as those that constitute the autism phenotypes.” In fact, predictive ability appears to be the main compromised component in autism. An impaired prediction leads to an impaired online object’s position estimation as well as a weak anticipation of the others’ actions. The perceptual awareness of others’ motor intentions conveyed in body movement or eye gaze is notoriously disrupted in autism (Pierno et al., 2006; Cattaneo et al., 2007). According to this approach, the stereotypical movements themselves would be configured as the expression of a prediction problem. Individuals with autism, through the repetitiveness of their stereotypes, would be able to have the sensation of controlling the surrounding environment (Sinha et al., 2014).

Thus, if motor abnormalities in ASD are derived from a predictive and perceptual problem, it is possible that its effects are observable from the first months of life. Several studies in which home videos were used to observe children before the age of two and then diagnosed as autism have found motor differences compared with typically developing children (Adrien et al., 1993; Teitelbaum et al., 1998; Baranek, 1999). This would mean that motor deficits could be present even before communication or social interaction problems, suggesting that motor impairment could actually be the precursors of the main symptoms of ASD (Leary and Hill, 1996; Nayate et al., 2005). During a critical developmental step, an ineffective perception of the external environment and an ineffective spatial interaction can certainly affect the interaction with the physical and social world, leading to the typical manifestations of autism. These findings highlight the need for further studies of motor difficulties as distinctive for ASD.

However, the literature still reports controversial results due to the weak methodological strategy and to the high variability of the autistic symptomatology. Although it is common to recognize the presence of motor impairment through interviews with parents, it is not easy to recognize these problems in childhood. For this reason, there is a growing need for an objective system to recognize autistic motor signatures from their early evidence. In recent years, several studies focused on using machine learning systems to recognize autism motor patterns.

In the present study, we try to determine whether a simple dragging movement on a tablet screen could be useful to accurately classify children with ASD. We developed a supervised machine learning system to discriminate children with ASD from typically developing children by means of kinematics analysis.

Taking advantage of a type of technology widely used in daily life and integrating it with classic diagnostic and assessment tools, we tried to enhance the assessment processes in autism. We aimed to make these processes more detailed and capable of providing an objective measure of the disorder. At the same time, we made the assessment sessions more motivating for the users and easy to administer for clinicians (Milano et al., 2017; Simeoli et al., 2019a).

## Related Works

In the last 20 years, authors have raised the problem of being able to categorize and recognize motor abnormalities in autism, taking advantage of new technologies and new methods of artificial learning. In particular, they focused on the recognition and anticipation of stereotypical motor movements (SMM) (Westeyn et al., 2005; Albinali et al., 2009, 2012; Min and Tewfik, 2010; Goodwin et al., 2011, 2014; Goncalves et al., 2012; Rodrigues et al., 2013; Großekathöfer et al., 2017; Milano et al., 2019). Using a variety of different features and semi-supervised classification approaches (orthogonal matching pursuit, linear predictive coding, all-pole autoregressive model, higher order statistics, ordinary least squares, and K-VSD algorithm), recognition rates of 86/95% for SMM and no-SMM have been documented. Such studies have had a great impact on rehabilitation and intervention in autism.

An important goal to reach is still related to the diagnostic and assessment processes. In fact, many attempts have been made to make predictions by recognizing and categorizing the typical motor patterns of ASD. The recent identification of motor disorders in young children who develop ASD represents a new goal for the development of early assessment tools (Trevarthen and Delafield-Butt, 2013; Licari et al., 2020). Crippa et al. (2015) used a supervised machine learning method to determine whether a simple upper-limb movement could be useful to recognize autism. They compared typically developing children and autistic children by means of kinematic analysis, reaching a maximum classification accuracy of 96.7%. Torres and Jose (2012) proposed to use a sophisticated measurement tool and statistical metric to classify and diagnose individuals with ASD (Torres and Jose, 2012).

Some other researchers have tried to delve into the topic by analyzing the coordinates of movement during the performance of simple tasks that required drag movements on the screen of a tablet. Anzulewicz et al. (2016) used an iPad gameplay and 262 features of movements, derived from touch screen and inertial sensors, and they showed that children with autism could be identified with up to 93% accuracy. Differences between children with ASD and typically developing children have emerged in terms of linearity, speed, and pattern of interaction (Simeoli et al., 2019b, 2020). Also, a greater engagement in carrying out cognitive tasks using digital tools has emerged, especially for individuals with severe autism (Simeoli et al., 2019a). Thus, it would appear that measures of motor patterns could provide a means to assess young children for autism.

Since several interactions of motion variables could, actually, affect the presumed typical autistic motor pattern, we cannot assume that there must necessarily be movement variables typical of autism and different in typically developing individuals. Thus, in this context, using classical statistical analysis can often be a stretch. For this reason, the aforementioned studies and the present one choose a predictive method rather than exploratory to address this issue (Yarkoni and Westfall, 2017).

In this study, we developed a software tool that, through a smart tablet device with touch screen sensor technologies, records kinematics movement while students are engaged to perform cognitive tasks. We extracted 12 features of movement, analyzed by a supervised machine learning method to obtain an automatic classification system, able to differentiate typical patterns of movements and autistic ones. This study aims (i) to describe motor information data that could differentiate children with autism from typically developing children and (ii) to develop a computational model that could recognize these motor patterns within ASD and typically developing children, in order to enhance autism assessment processes.

## Materials and Methods

### Participants

The study was attended by 60 children aged between 5 and 10 years, divided into two groups: 30 children with an average age of 7 years, standard deviation 1.4, clinically diagnosed with ASD according to the Diagnostic and Statistical Manual of Mental Disorders (5th ed.); and 30 children, aged 6 years and 8 months, standard deviation 1, with typical development (TD). The original version of the Leiter-3 International Performance Scale was used to assess the IQ for both groups. The IQ score for the TD group ranged between 74 and 110, and the ASD group covered a range from 59 to 109. Six children in the ASD group had a mild mental retardation with an IQ score ranging between 59 and 70 (World Health Organisation [WHO], 2016). No moderate, severe, or profound mental retardation was detected.

All participants had normal vision and no sensory or motor deficit. Any child whose clinician or teacher was uncertain about the child’s diagnosis or health was excluded.

The ASD participants were recruited from the Neapolisanit S.R.L. Rehabilitation Center. Inclusion criteria were as follows: a diagnosis of autism according to both DSM-V clinical criteria and to the Autism Diagnostic Observation Schedule (ADOS-2) (Lord et al., 2012), age range between 5 and 10 years, and no existing comorbidities. The TD participants were recruited from a primary school. Exclusion criteria were suspected signs of autistic spectrum disorders, developmental abnormalities, and current or past history of psychiatric or neurological disorders.

All the participants belonging to the ASD group were diagnosed with ASD by qualified doctors and professionals in the sector. They have no affiliation with our laboratory or our research. Children with ASD follow psychomotor and speech therapy treatment at the Neapolisanit S.R.L. Center. No specific comorbidity was reported.

Prior to the study, children’s parents gave written informed consent for their children’s participation in the study. The experimental protocols employed were approved by the Federico II University of Naples Ethical Committee of Psychology Research.

### Materials

**FIGURE 1:**
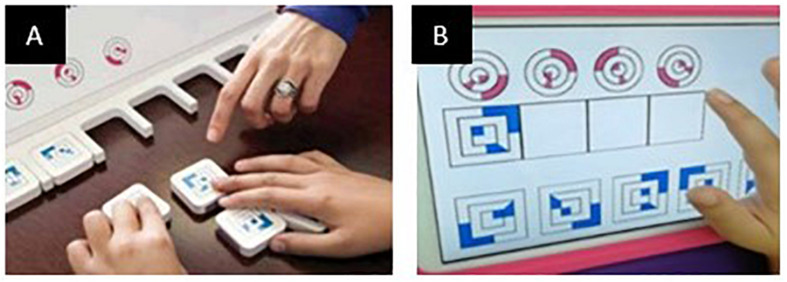
Panel **(A)** depicts the Leiter-3 test in its original version; on the right, panel **(B)** is an example of a test scene in its digital version.

The movement detection software was developed in Unity and C#. The study was performed on Android tablet 6.0, size (*H* × *W* × *D*) 241.9 × 149.5 × 8.5 mm, screen size 9.6 inches with a resolution of 1,280 × 800 (WXGA) and a refresh rate of 60 Hz. The tasks were presented to the children within a bespoke app organized in a sequence of scenes which play tasks of the cognitive battery of the Leiter-3 test (Roid et al., 2013; Figure 1), a totally nonverbal test of intelligence and cognitive abilities, widely used in ASD individuals. The software plays exactly the same tasks of the Leiter-3, presented in the same order, and the administration of the digital version followed the same rules of the original test. Participants were required to perform the tasks according to their cognitive abilities. The examiner switched from a subtest to another, according to the instruction procedure of the original version of the test, after the error threshold was reached.

Scenes are composed of a maximum of five moving cards and eight fixed images (the placeholders). Moving cards are placed at the bottom of the screen and they can be dragged across the screen; placeholders are placed at the top. The placeholders are programmed to catch the moving cards dragged nearby. For each task, placeholders and moving cards range from a minimum of two to a maximum of eight and can include distractors.

The battery is composed of five different subtests related to five different cognitive domains. Each subtest is composed of 10 or more items. Tasks are divided in five cognitive categories as follows: (a) figure-ground (FG) tasks require to identify parts of figures within a complex stimulus. The user must identify the correct areas, within a complex picture, where to place the moving cards representing parts of the picture above. The placeholders are positioned within the target image and are invisible to the user who must match identical figures within the complex background; (b) figure completion (FC) demands the ability to recognize an entire object from all its roles randomly arranged on the scene; (c) classification analogies (CA) requires classification of objects or geometric figures in which participants have to complete a sequence of geometric shapes and matrices with increasing levels of complexity; (d) sequential order (SO) requires to place figures according to a logical, SO; and (e) visual patterns (VP) requires discrimination and matching of pictures. All the subtests, except FG, are arranged in the same way. The users have to drag the moving cards at the bottom into delimited placeholders positioned at the top of the screen.

Tasks are presented following an increasing level of complexity and are characterized by a progressive increase of distracting stimuli and details of the images. The ascending level of complexity requires increasing levels of attention and decision-making.

Since all participants correctly performed at least the first five items of each subtest, only the trajectories derived from these items were analyzed.

### Experimental Protocol

**FIGURE 2:**
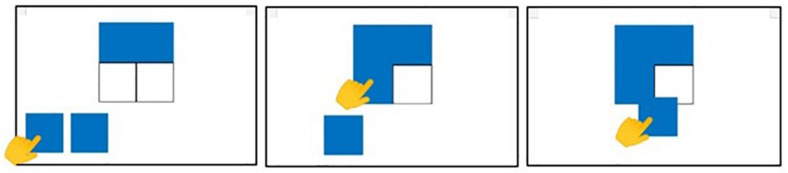
An example of dragging movement during the performance of a CA task. The users are required to drag the moving cards from the bottom to the corresponding placeholders at the top of the screen, according to the task demand.

During the study, participants sat in front of a table 65–70 cm high according to the age of the child. The experimenter sat at the opposite side of the table. Children performed the task on the Android tablet placed on the table in front of the child within 20 cm of the edge of the table. At the beginning of each subtest, the examiner provided the instructions to carry out each task, according to the instruction procedures of the original version of the test. The experimental task consisted of dragging images on the tablet screen from a point to another, according to the cognitive demand of each specific task (Figure 2). After the instructions, the examiner left the child free to perform the task without any further aid. The instruction phase included a series of guides that encouraged attention to the main cognitive target, using pointing and specific gestures, without any vocal aid. If needed, the examiner can demonstrate how to carry out the task by moving the cards himself. This is allowed only for the first item of the first subtest (FG1). Coordinates extracted from this item have not been considered for the analysis.

Switching between tasks was automatic when the child completed the task. The task was considered complete when each of the moving images was placed into one of the placeholders at the top, regardless of the performance result. In case the child did not place all the moving cards above the placeholders, the examiner switched to the next by double clicking an invisible button placed at the upper corners of the screen.

### Data Acquisition and Analysis

#### Features Extraction

The software recorded information about the position over time of each stimulus displayed on the screen during each task and, at the same time, information about the participant’s finger position. Touch data were collected runtime at a rate of 40 Hz. That is, the software recorded 40 pair coordinates (*x*, *y*) of movement per second. Information about which task was performed was always associated with the touch data. The recorded space-time touch data were analyzed as trajectories of movement. We considered as “finite” trajectories all the coordinates resulting from the dragging movement from the first tap on the screen until the finger was lifted from the screen at the end of the dragging movement. For each “finite” trajectory, we obtained the value of the features in Table 1. For the analysis, we used the average value merged per task.

**TABLE 1:** Features obtained from the RStudio traj package analysis.

Feature name	Description
MeanSpeed	Average speed values per task
MaxSpeed	Average value of the maximum speed peaks reached during the performance of a task
MinSpeed	Average value of the minimum speed peaks reached during the performance of a task
sdSpeed	Standard deviation of the speed values collected during the task
MeanAcceleration	Average acceleration values per task
MaxAcceleration	Average value of the maximum acceleration peaks reached during the performance of a task
MinAcceleration	Average value of the minimum acceleration peaks reached during the performance of a task
sdAcceleration	Standard deviation of the mean acceleration values collected during the task
STH	The Straightness index as ratio between the distance of the starting and ending points of a trajectory and its length
DC	Directional change is the change in direction over time
sdDC	Standard deviation of directional change value obtained during the task
MeanLength	The average amount of finite trajectories conducted during each task

*STH is a number ranging from 0 to 1, where 1 indicates a straight line. STH is an index of linearity. DC is defined for each pair of steps so that a trajectory may be characterized by the mean (DC) and standard deviation (sdDC) of all directional changes. DC may be used as an index of nonlinearity, and sdDC as a measure of irregularity.*

Seventeen variables were extracted from the analysis of these trajectories: 12 features refer to characteristics of the trajectories (Table 1), and five additional variables are related to the task during which those features have been recorded, in order to provide implicit information about the difficulty level of the task performed. The features allowed us to obtain a comprehensive computational description of a child’s motion patterns during the interaction with the device.

Features were computed from the consecutive sets of raw coordinates using RStudio software and the *traj* package (Leffondree et al., 2004; Sylvestre et al., 2006). Motion data for each task were aggregated and divided into finite trajectories based on the start and stop of each particular movement. The analysis was conducted, and features were extracted for each of these trajectories. All these data were then aggregated in order to find the average values for each task. The final dataset consisted of the mean value for all the features (Table 1) divided per task (five difficulty levels).

Two types of information were obtained: (i) kinematics information, e.g., speed or acceleration, and (ii) touch-based functions, e.g., the number of trajectories drawn during the task and the average length.

These compiled datasets were entirely used as input for an artificial neural network (ANN) (see below). The dataset included information about the tasks, as per Leiter-3 structure, in order to classify the motion pattern according to the cognitive demand required during the movement. Five different cognitive domains within the Leiter-3 scale were identified and analyzed.

#### Classification Methods

The ANN was used to recognize the autism motor signatures, since the capacity of ANNs to process complex and nonlinear relationships between variables is well known (Hornik et al., 1989; Chen et al., 1990). Seventeen features were obtained, as explained above. The set of data was composed of the average values of each feature, divided into tasks. Data were labeled accordingly to the child’s diagnostic group (ASD or TD). Data were standardized, before being assigned to the ANN.

The ANN used for the ASD/TD classification was a feedforward multilayer perceptron, composed of an input layer of 17 neurons, an output layer of two neurons, and a hidden layer whose number of neurons has been selected through grid search optimization with cross-validation, in a descending search starting from 10 hidden neurons, as we tried to keep to the model as simple as possible in order to reduce overfitting. Indeed, it is a well-known result of ANN, the fact that simple models, i.e., with few hidden units, are less prone to overfitting (Musavi et al., 1994).

The tangent hyperbolic (*Tanh*) activation function was used for the five neurons in the hidden layer. *Tanh* is widely used for the hidden layers of an ANN. Its values are between −1 and 1, and the average turns out to be 0 or very close to it; in this way, it helps to center the data by bringing the average close to 0. For the output layer, a normalized exponential function (*Softmax*) was used. The *Softmax* function will output a probability of class membership for each class label and attempt to best approximate the expected target for a given input. Adaptive moment estimation (*Adam*) learning algorithm was used to update the iterative network weights based on the training data (Kingma and Ba, 2015), and for the training, we used a sparse categorical cross-entropy loss function to calculate the model error.

To evaluate our approach and select the best architecture, we use a 10-fold cross-validation with five repetitions: Using the 10-fold cross validation scheme, the dataset was randomly divided into 10 equal subsets. At each run, nine subsets were used to construct the model, while the remaining subset was used for prediction. The average accuracy for the 10-fold was recorded as the final measurement.

To eliminate the statistical variations due to the random weight initialization, we repeated the resampling procedure five times and recorded the average classification errors.

The dataset was composed of a total of 1,500 samples, coming from 60 subjects doing 25 tasks divided into five subtasks, and the dataset is randomized between subjects and tasks. Each sample was composed of 12 motor-based features and five task features, by which the current task was equal to one and the others equal to zero. The target was a simple two class one-hot encoded dataset where ASD subjects were assigned to (1,0) and TD to (0,1).

Data regarding the motor features were standardized. The general method of calculation is to determine the mean distribution and standard deviations for each feature. Next, we subtract the mean from each feature. Then, we divide the obtained values of each feature by its standard deviation.

Then, the 10-fold cross-validations with five repetitions are applied to a 500-epoch lasting training process, and for each fold, we take the 10% of the samples for testing and the remaining 90% for training, regardless of the number of ASD or TD subjects in the test or training set.

The purpose was to generate a model able to learn from the selected characteristics how to discriminate individuals belonging to two different groups and correctly classify, through these characteristics, new unlabeled individuals.

Moreover, in order to understand how the kinematic and touch features provided as input for the ANN contributed to the classification, a form of sensitivity analysis is applied to the model, where the accuracy, sensitivity, and specificity of the model are calculated over the whole number of features ranging from 1 to 12 (subtest features were always provided to the ANN) in an iterative way. In particular, we have iteratively applied the method called *Improved stepwise selection 1*, as presented in Olden et al. (2004). It assesses the change in the accuracy, sensitivity, and specificity of a trained ANN by sequentially removing input neurons from the neural network. The resulting changes for each variable removal illustrate the relative importance of the predictor variables (see Gevrey et al., 2003). In our modification, starting from the entire set of 12 input variables, we selectively remove one single input neuron and record the change in accuracy, sensitivity, and specificity after the removal of every input variable, one at a time. That is, we test all possible 12 variables. Once the variable with the lesser impact on the overall performance of the ANN is identified, it is permanently removed. The same process is applied to all remaining input variables, until the process exhausts the number of input variables of the model. In this way, it is possible to measure the relative importance of independent variables for the final categorization performance of the one neural network found as the best predicting model.

## Results

The grid search with 10-fold cross-validation, applied to set the most effective number of hidden neurons for the ANN, indicated comparable accuracies for models from 10 to 5 hidden neurons. Learning rate was set to 0.01 and synaptic weights were initialized following the so-called *Xavier initialization* (Glorot and Bengio, 2010). The accuracy started to degrade with fewer than five hidden neurons. To keep the model simple, we have chosen the ANN with the smaller layer of hidden units that showed the best accuracy as the final model, that is, the ANN with five hidden units.

**FIGURE 3:**
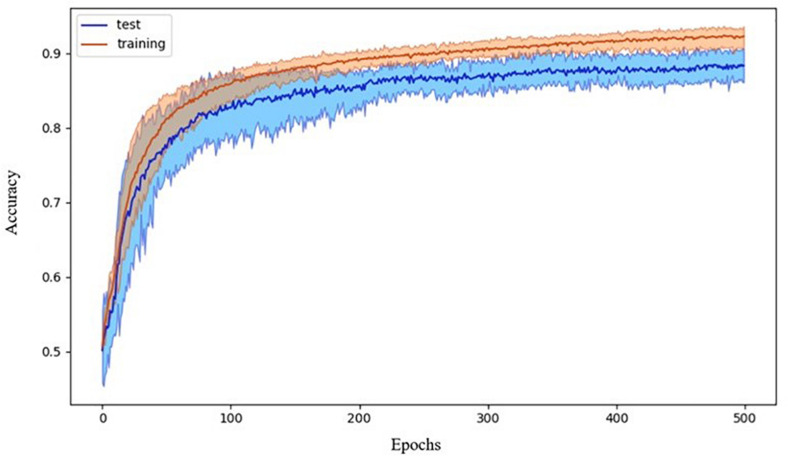
Accuracy during the training process. Data refer to the average accuracy of all the models of the 10-fold CV with five repetitions. Mean and 90% bootstrapped confidence intervals of the mean (shadow area) across all the replications.

The chosen ANN was able to successfully classify participants by diagnosis. The 10-fold cross-validation showed an accuracy of 88 ± 3%. Data showing the accuracy versus the epoch for the 10 cross-validation models are reported in Figure 3.

**FIGURE 4:**
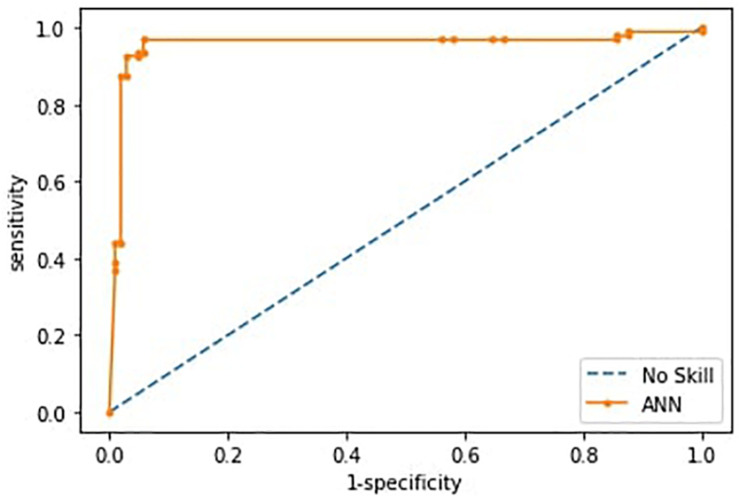
Receiver operating characteristics curves (ROC) of the ANN model. The curve is derived from the sensitivity and specificity index, the rate of correctly classified samples in the positive and negative classes.

Having checked that no overfitting is present in every fold, for the successive analysis, we used the best trained model across the 10-fold, as reported in Riccio et al. (2020). Such ANN correctly differentiated individuals within ASD and TD groups with an accuracy of 0.93 (sensitivity 0.87, specificity 0.98). The ROC curve is shown in Figure 4.

**FIGURE 5:**
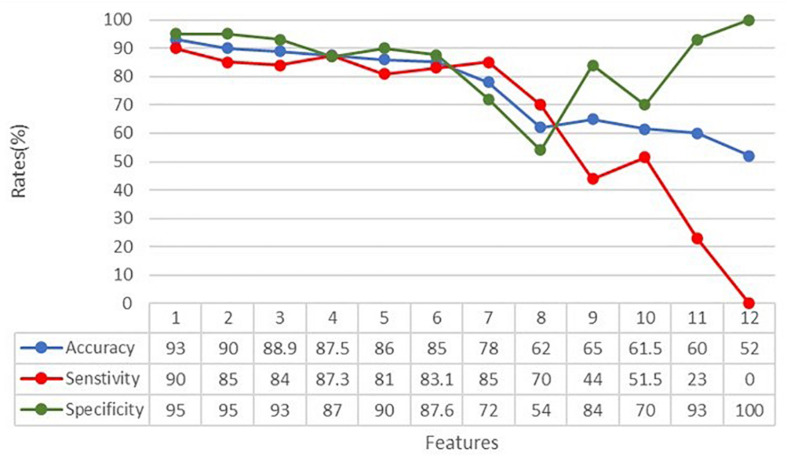
Classification accuracy, specificity, and sensitivity rates in relation to the number of features analyzed by the ANN. Features have been withdrawn as follows: (1) sdAcc, (2) STH, (3) sdSpeed, (4) sdDC, (5) MaxSpeed, (6) DC, (7) MeanRow, (8) MinSpeed, (9) MeanSpeed, (10) MinAcc, (11) MaxAcc, and (12) MeanAcc.

In order to understand how the input variables contributed to the classification, the accuracy, sensitivity, and specificity rates were calculated over the 12 input variables representing the kinematic and touch measures, as specified above. The subtest features were always provided to the ANN. Figure 5 shows the dependence of the metrics on the number of considered features. From the graph and the accompanying table, we can see that accuracy, specificity, and sensitivity rates reached their maximum when considering all the extracted features. Moreover, according to the iterative assessment described above, the metrics degrade slowly until the seventh variable is removed (78% accuracy). After that, the accuracy falls sharply until 52% of accuracy is reached.

According to their progressively greater impact on the overall classification performance, input variables have been withdrawn as follows: (1) sdAcc, (2) STH, (3) sdSpeed, (4) sdDC, (5) MaxSpeed, (6) DC, (7) MeanRow, (8) MinSpeed, (9) MeanSpeed, (10) MinAcc, (11) MaxAcc, and (12) MeanAcc. It means, for example, that variables number 1, 2, or 3 have less impact on the classification than variables number 10, 11, or 12, indicating that variables related to speed and acceleration of the finger seems more important than other measures, such as the straightness (STH) and the coherence (DC) of the entire trajectory.

## Discussion

Autism is primarily assessed by relying on qualitative judgments by expert clinicians and through semi-structured interviews conducted by parents and caregivers. Given this gold standard, in recent years, the use of a pattern recognition method has obtained great attention as a suitable tool to define objective, quantitative measures of the disorder.

The purpose of the present work was to use kinematic features of simple dragging movements as predictors to discriminate children with ASD from typically developing children. Our results suggested that motor patterns related to autism can be identified by machine learning method. Our analysis showed that 17 features were sufficient to classify autism with an accuracy rate of 93%, sensitivity of 87%, and specificity of 98%.

The study shows that autism can be identified by the interaction of a few specific movement features and their characteristics. We cannot assume that these features are statistically different between groups, but we can suppose that the dynamic interaction of these features can be categorizable.

**FIGURE 6:**
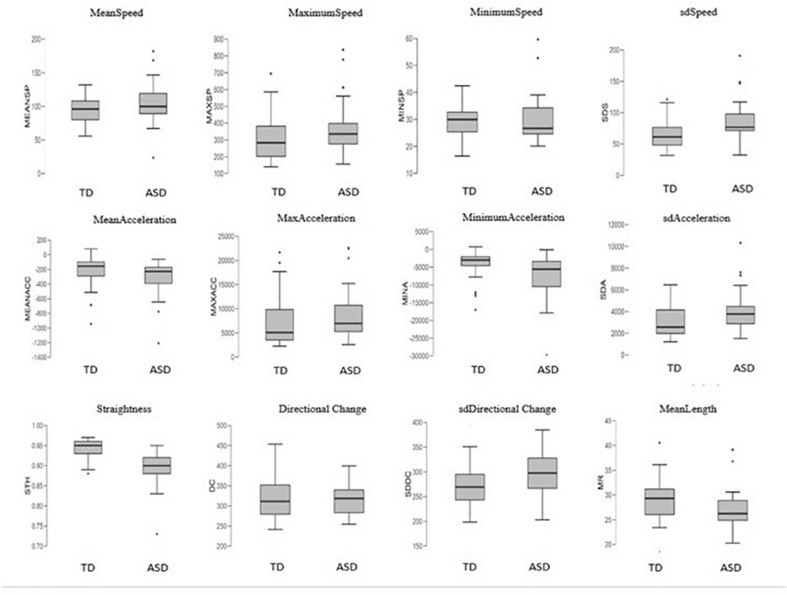
Boxplots of the 12 kinematics features extracted from coordinates of movement and compared between the groups. Features definition in Table 1.

In order to understand how these features appear between groups, we observed them in detail (Figure 6). Results revealed that autism motor patterns are characterized by low linearity of movements. As shown in Figure 6, the ASD group reached a low level of STH and high level of DC. This means that their trajectories were not straight and characterized by many changes of direction. Furthermore, the average length of their trajectories (MeanLength) was lower than that of the TD group, indicating more fragmented movements. Velocity index revealed a wide range of values associated with speed and acceleration of ASD children’s movements. In fact, they showed big values of MeanSpeed and MaximumSpeed, but low values of MinimumSpeed. Likewise, mean gesture acceleration also covered a wide range for the ASD group, with great MaximumAcceleration value and low MinimumAcceleration. These results explain the higher standard deviation values (sdSpeed; sdAcceleration) shown for both speed and acceleration in the ASD group (Figure 6).

Our results are consistent with the findings of Anzulewicz et al. (2016), since they found that ASD children displayed greater force of impact and different patterns of force than their typically developing peers. The authors explain these characteristics as likely due to maintaining great velocity at contact with consequent increased impact force. These findings are in line with the notion that prospective guidance of goal-directed movement is disrupted in ASD, and this disruption could likely determine over- and undercompensations during the movement, e.g., during the movement to reach the tablet as shown by Anzulewicz et al. (2016) and during the dragging movement across the screen as shown in our study. Other features contributed to describe this phenomenon as, e.g., for the results related to DC, STH, and MeanLength of trajectories. In fact, our results indicated that autism finite trajectories were basically very short, as shown in Figure 6 for the MeanLength feature. In addition, our autistic trajectories were characterized by a low straightness and linearity and a great irregularity, as indicated by the values of STH, DC, and its SD, respectively (Figure 6). All these characteristics could be linked to the compensations mechanism described above.

Our results could be considered in line with the previous findings about optical motion tracking experiments of goal-directed tasks. Considering the motion structure described above, it could suggest that individuals with autism make greater moment-by-moment adjustments of their progressive movements compared with the neurotypical group (Cook et al., 2013; Torres et al., 2013; Whyatt and Craig, 2013). Whyatt and Craig (2013) demonstrated that ASD children, during their movements, made multiple corrective movements, reaching greater velocity at the end of their motion. Our study confirms these findings reporting a general greater velocity and acceleration for children with autism, but also describing the movement as more fragmented and less straight. The higher rate of change of direction could be a representative index of the aforementioned overcorrection of movements.

These results support the idea of the presence of a fundamental deficit in the prospective control of movement (Klin et al., 2003; Fabbri-Destro et al., 2009; Trevarthen and Delafield-Butt, 2013; Lawson et al., 2014). This deficit would manifest itself with the interruption of the anticipatory, or feedforward mechanism (Mari et al., 2003; Papadopoulos et al., 2012) or feedback re-afferences (Torres et al., 2013), during the goal-directed actions. Deficits in perception of others’ intentions and on fluid selective attention on the adequate stimuli to program a consistent movement with the external environment are notoriously damaged in autism (Pierno et al., 2006; Cattaneo et al., 2007). All these findings support the idea of a deficit in sensorimotor timing integration that affects the perception–action process and the ability to understand the social environment. If the proprioceptive feedback that allows online movement guidance is interrupted, movement control errors can be generated, resulting in abnormal motor signature that we are proposing to use as markers for children with ASD.

About index and markers for identifying autism, many studies have focused on discovery biomarkers of the disorder, but the heterogeneity and the complex etiology of autism have always made this process very tough. For this reason, studies of recent years and our study are focusing on identifying computational biobehavioral markers of the disorder. However, the analysis of these markers requires further study to avoid potential attribution errors. In fact, the motor signature identified may overlap with other disorders, such as attention deficit disorder, motor coordination disturbance, or general intellectual disabilities. Further studies are needed to elucidate this aspect.

The present study is a theoretical demonstration of the development of accessible and attractive assessment tools that can integrate new important information to the ordinary assessment process for autism.

Despite our promising results, some methodological limitations are evident. One of the limitations is certainly related to the small sample size, and a replication on a larger sample is needed to validate this method on a new not trained dataset. Further studies are necessary to test whether the algorithm used could remain predictive also for a greater sample or if it requires to be retrained.

Furthermore, we were not able to exclude intelligence as confounders. Even if we tested children’s IQ through the test tasks themselves, we did not use IQ as an independent feature for the analysis. However, most of the ASD children who participated were classified as high functioning and only six of the participants with ASD had a mild mental retardation with an IQ between 59 and 70. In order to reduce the cognitive interference, we selected for the analysis the features extracted from tasks correctly performed by all participants.

Our study involved children with different types of autism (from high to low functioning) since the hypothesis was that autism, regardless of type, could affect the classification.

Previous studies assumed the presence of a sensory integration dysfunction (SID) to explain motor abnormalities in autism (Klin et al., 2003; Mari et al., 2003; Fabbri-Destro et al., 2009; Papadopoulos et al., 2012; Torres et al., 2013; Trevarthen and Delafield-Butt, 2013; Lawson et al., 2014). SID is not currently recognized as a distinct medical diagnosis, but it is usually found in development conditions, particularly in autism. Other conditions could be affected by SID as for example ADHD. Thus, in addition to an IQ control, future extensions of this work should include other neurodevelopmental disabilities in order to verify the specificity of these motor signatures for ASD.

## Conclusion

In conclusion, this study represents a proof of concept that kinematic analysis of a simple dragging movement can be useful to discriminate individuals with autism and differentiate them from their typically developing peers. The predictive power of our algorithm might support clinical assessment processes and encourage a computer-aided diagnosis perspective.

Our future aim is to recognize these autistic signatures in younger children and, thus, facilitate the diagnostic processes.

However, we can affirm that the automatic learning of autistic motor patterns, through kinematic analysis, during tablet cognitive assessment, can be considered a promising new method for autism detection and that it could enable the use of biobehavioral markers for the assessment of the disorder. Through this study, we also suggest how technologies, integrated with classic diagnostic and clinical tools, can be wisely used to support the clinic and intervention in the field of ASD, facilitating and refining the research, diagnosis, and assessment processes.

## Data Availability Statement

The raw data supporting the conclusions of this article will be made available by the authors, without undue reservation.

## Ethics Statement

The studies involving human participants were reviewed and approved by the Ethical Committee of Psychological Research of the Department of Humanities of University of Naples Federico II. Written informed consent to participate in this study was provided by the participants’ legal guardian/next of kin.

## Author Contributions

RS contributed to the conception and design of the study, developed the software and carried out the experimental sessions, contributed to the data analysis, and drafted the manuscript. NM contributed to the data analysis developing the machine learning model. AR provided substantial contributions to the acquisition of data and coordinating the experimental work. DM supervised the work, contributed to the conception and design of the study, and coordinated the data analysis and software development process. All authors read and approved the final manuscript.

## Conflict of Interest

The authors declare that the research was conducted in the absence of any commercial or financial relationships that could be construed as a potential conflict of interest. The reviewer, GT, declared a shared affiliation, with no collaboration, with one of the author, NM, to the handling editor at the time of the review.
